# Plant Associated Rhizobacteria for Biocontrol and Plant Growth Enhancement

**DOI:** 10.3389/fpls.2021.634796

**Published:** 2021-03-17

**Authors:** Xiurong Jiao, Yoko Takishita, Guisheng Zhou, Donald L. Smith

**Affiliations:** ^1^Institute of Agricultural Science and Technology Development of Yangzhou University, Yangzhou, China; ^2^Joint International Research Laboratory of Agriculture and Agri-product Safety, The Ministry of Education of China, Yangzhou University, Yangzhou, China; ^3^Department of Plant Science, Faculty of Agricultural and Environmental Sciences, McGill University, Montreal, QC, Canada

**Keywords:** biocontrol, biocontrol agents, plant growth promoting rhizobacteria, dual benefit, phytomicrobiome

## Abstract

Crop disease remains a major problem to global food production. Excess use of pesticides through chemical disease control measures is a serious problem for sustainable agriculture as we struggle for higher crop productivity. The use of plant growth promoting rhizobacteria (PGPR) is a proven environment friendly way of controlling plant disease and increasing crop yield. PGPR suppress diseases by directly synthesizing pathogen-antagonizing compounds, as well as by triggering plant immune responses. It is possible to identify and develop PGPR that both suppress plant disease and more directly stimulate plant growth, bringing dual benefit. A number of PGPR have been registered for commercial use under greenhouse and field conditions and a large number of strains have been identified and proved as effective biocontrol agents (BCAs) under environmentally controlled conditions. However, there are still a number of challenges before registration, large-scale application, and adoption of PGPR for the pest and disease management. Successful BCAs provide strong theoretical and practical support for application of PGPR in greenhouse production, which ensures the feasibility and efficacy of PGPR for commercial horticulture production. This could be pave the way for widespread use of BCAs in agriculture, including under field conditions, to assist with both disease management and climate change conditions.

## Introduction: The Current Situation Of Plant Disease

In recent decades, crop productivity has been challenged by threats from both plant diseases and large inputs of artificial pesticides, including those applied to deal with the disease challenge. Crop yield reductions due to plant diseases are often in the range of 21–30% globally in some major crops (Savary et al., 2019). At the same time, many plant pathogens have evolved resistance to long-used chemical control measures (Lucas, 2011). As a result, some plant diseases of economic importance have become more difficult to control, principally due to the lack of effective compounds (Bailey, 2010); intensive crop production practices and food market globalization have aggravated this situation (Fones et al., 2020). Many chemical pesticides are not readily broken down into simple and safer constituents, and as a result, they remain in soil as toxic residues, sometimes with associated human health issues (Gilden et al., 2010). Furthermore, increasing public awareness of environmental and health issues associated with synthetic chemicals is causing a shift toward more sustainable crop management practices that rely less heavily on synthetic chemicals (Chandler et al., 2008; Donley, 2019). Given this situation, heavy use of synthetic agrochemicals has come to be considered an unsustainable approach, and new and sustainable agricultural practices have become a focus of modern agriculture research. Increasing demand for alternatives to pesticides has provided opportunities for expanded application of biological control (Barratt et al., 2010; Gay, 2012).

From environmental and health perspectives, plant growth promoting rhizobacteria (PGPR) have been proposed as one of the most promising alternatives to synthetic chemicals. However, apart from the points that mentioned above, crop production has always being influenced by climatic conditions and more concerningly and profoundly as climate change conditions develop. Under climate change conditions, plant pathogen control will also experience new challenges (Chaloner et al., 2020), including more difficulty in controlling current disease; combating new emergence of pathogens (McDonald and Stukenbrock, 2016); meeting the challenges associated with shifting geological distributions of pathogens (Bebber et al., 2019; Fones et al., 2020). Especially for the seemingly promising biological disease control methods, as their efficacy can be strongly affected by environmental conditions. Under these circumstances, researchers need to explore a strategy for future food production that includes consistent and sustainable crop disease control.

## Pgpr As Promising Biocontrol Agents

Soil is both a source of nutrients for plant growth and a complex ecosystem where bacteria, fungi, protists, and animals live in diverse and active/coordinated communities (Müller et al., 2016). Many plant-associated soil/root microbes (the phytomicorbiome) have established relationships with plants that can be competitive, exploitative, or neutral; the plant plus the phytomicrobiome form the holobiont (Lyu et al., 2020). Recently, researchers have begun to investigate the possibility of both alleviating pathogenic effects and promoting plant growth *via* beneficial rhizobacteria (Zhang et al., 2013; Qiao et al., 2017).

Plant roots host an enormous range of microbial organisms (Bulgarelli et al., 2013) and an important, the next step is to determine which rhizobacterium, or combination of rhizobacteria, most benefit the host plant. Developing selective pathogen control agents directed at the target plant species could be a key aspect of this (Lommen et al., 2019). Microbial strains that have co-evolved with plants for protracted periods of time, and promote plant growth, are likely to provide more than one benefit, such as pathogen control (Smith et al., 2015; Kruitwagen et al., 2018). Rhizosphere microbiomes differ among plant species (Turner et al., 2013; Zgadzaj et al., 2016); plants exert control over the composition of their microbiomes (Smith et al., 2015; Zgadzaj et al., 2016). As a result of their long co-evolution with plants, microbes can indirectly influence plant phenotypic plasticity and plant health *via* modulation of plant development and defense responses (Goh et al., 2013).

A wide range of microorganisms, inhabit the rhizosphere, comprising a tremendous source of PGPR (Antoun and Kloepper, 2001). The plant-beneficial members of the phytomicrobiome, the community of bacteria that colonize the rhizosphere, on the root surface, or in the spaces between cells of the root cortex or the root cells themselves (Gray and Smith, 2005; Inui Kishi et al., 2017). PGPR have been co-evolving with associated plants since plants colonized terrestrial environments, leading to development of synergistic interactions with the host plants (Gouda et al., 2018). There have been a large number of publications around research investigating the effects, mechanisms, and potential for successful application of PGPR to production of crop plants grown under controlled environment conditions. This is very important for the development of more widespread biological control approaches, including field conditions.

For vegetable production, quality control and safety are more important and have a close relationship with human health as we often consume them less processed or unprocessed. It is much easier to deploy PGPR in greenhouse production systems, as the environmental conditions are controlled; at the same time, there is a large number of potential BCE strains identified and potentially available for possible deployment (Singh et al., 2017); these are proven to be effective in greenhouse experiments (Zhang et al., 2010; Hahm et al., 2012; Lamsal et al., 2012; Liu et al., 2018). For example, *Bacillus* spp. has become an significant microbe for disease suppression under field conditions (Miljaković et al., 2020) and *Pseudomonas fluorescens* has also been considered as a promising biocontrol agent (BCA; Panpatte et al., 2016). Several specific isolates – *Pseudomonas stutzeri*, *Bacillus subtilis*, and *B. amyloliquefaciens* were identified and proven to be successful in root colonization and to have significant suppression of the pathogen *Phytophthora capsici* during cucumber plant growth (Islam et al., 2016). *Penicillium* sp. and *Rhizopus stolonifer* cause fruit infections and were found to be effectively suppressed at the post-harvest stage by application of *Bacillus subtilis* (Punja et al., 2016). Isolates of *B. amyloliquefaciens* significantly inhibit *Fusarium* wilt disease, caused by *Fusarium oxysporum*, under greenhouse conditions (Gowtham et al., 2016).

These examples illustrate that PGPR, as BCAs, are effective under controlled environment conditions, providing strong support for application of PGPR in greenhouse production systems, which ensure the feasibility and efficacy of PGPR for commercial horticulture production.

Prevention of pathogen infection and promotion of plant growth under abiotic stresses are both indirect mechanisms for PGPR, which should not be separated. Furthermore, for practical application, PGPR with biocontrol effects would be more valuable if they also promoted plant growth.

Climate change is one of the major causes for more severe abiotic stress: like salinity, drought, and cold. It has been illustrated that PGPR not only alleviate pathogen control and also help improve plant tolerance of abiotic stresses such as salinity (Ilangumaran and Smith, 2017; Numan et al., 2018; Bhat et al., 2020) and drought (Vurukonda et al., 2016; Jochum et al., 2019; Kour et al., 2020; Leontidou et al., 2020). Quite a few strains have proven to help combat stress and promote plant growth under conditions of abiotic (Dehghani Bidgoli et al., 2019; Goswami and Deka, 2020). However, most researchers have investigated PGPR under these environmental stresses; few have also examined their biocontrol efficacy, and particularly in conjunction with abiotic stress. This is an important consideration in biocontrol under field conditions, and as climate change conditions develop. However, if PGPR have coevolved with plants for protracted periods of time, it is likely that they provide more than one benefit to the host plant, and given that the plant provides them with energy (reduced carbon) and niche space, the provide benefit to themselves (Fan et al., 2020).

## Mechanisms For Biocontrol with PGPR

PGPR can directly promote plant growth by improving the uptake of certain nutrients from the environment (such as nitrogen fixation and phosphate mobilization) or production of phytohormones, like indole-3-acetic acid (IAA), gibberellic acid, or phytohormone regulators such as cytokinin and 1-amino-cyclopropane-1-carboxylate (ACC) deaminase (Vejan et al., 2016; Gouda et al., 2018). PGPR ability to produce phytohormone, associated metabolites, and signal compounds also explains their mitigation of abiotic stress conditions such as drought (Jochum et al., 2019) and salinity (Ilangumaran and Smith, 2017; Abbas et al., 2019). Another reason for this is that PGPR can modify root morphology, which often leads to increased root surface and so enhanced water and nutrient uptake (Kumar et al., 2019; Goswami and Deka, 2020; Nawaz et al., 2020). In addition, PGPR compete with other bacteria by colonizing quickly and obtaining more nutrients for themselves, restraining the growth of other organisms (Salomon et al., 2017).

In addition, pathogen controlling PGPR can alleviate or prevent the detrimental effects of one or more phytopathogenic organisms. The underlying mechanisms are diverse and rhizobacteria possess a wide range of mechanisms, one or several in combination, which are associated with specific host plants (Choudhary et al., 2011). The most widely recognized direct mechanisms for biological control are the suppression of pathogens through synthesis of anti-pathogen compounds, such as antibiotics (Raaijmakers et al., 2002), antimicrobial peptides, bacteriocins metabolites, toxins, and enzymes (Compant et al., 2005). Antibiotics are antimicrobial substances of low-molecular-weight; once released by PGPRs, they can prevent the growth or metabolic activities of other microorganisms (Duffy et al., 2003). They can suppress diseases by inhibiting pathogen cell walls synthesis, influencing membrane structures, and inhibiting the formation of initiation complexes on the small subunit of the ribosome (Maksimov et al., 2011). Bacteriocins (peptides with antimicrobial activity, such as polymyxin, circulin, and colistin) are proteinaceous toxins that are secreted by bacteria and which can destroy related/metabolically similar bacterial species by damaging the bacterio-cinogenic cells (Riley and Wertz, 2002; Abriouel et al., 2011; Nazari and Smith, 2020). Normally, they are strain targeted, and so only toxic to closely related bacteria. A large number of bacteria produce more than one bacteriocin, and some of them show broader spectra of inhibition (Abriouel et al., 2011).

Siderophores act as specific ferric iron chelating agents, especially under iron-limited conditions, making it unavailable to the phytopathogens and protecting plant health (Crowley, 2006; Shen et al., 2013). Some PGPR have been shown to enhance plant growth by producing extracellular siderophores, which help control of several plant diseases as siderophores deprive the pathogen of iron nutrition, thus inhibiting disease development (O’Sullivan and O’Gara, 1992; Sharma and Johri, 2003; Radzki et al., 2013).

Therefore, antibiotics, bacteriocins, and siderophores have been indicated as the three most effective mechanisms for identifying potential biocontrol effects before further evaluation *in vivo* (Kloepper et al., 1980). For this reason, production of siderophores and other plant-beneficial metabolites has been used in many studies when exploring PGPR as potentially functional plant disease control agents (Maksimov et al., 2011; Subramanian and Smith, 2015).

More indirectly, PGPR can enhance crop stress tolerance by synthesizing microbe-to-plant signal compounds. Signal compounds including phytohormones and specific signal compounds for both plant-to-microbe and microbe-to-plant communications (Lyu et al., 2020). This close relationship between the host plant and their “specific” phytomicrobiome members regulates aspects of growth and metabolism in both elements of the holobiont (plant and phytomicorbiome). Lipo-chitooligosaccharides (LCOs) and thuricin 17 are two microbe-to-plant signals that have been found to increase stress tolerance in a wide range of plant species (Smith et al., 2015; Subramanian et al., 2016; Lyu et al., 2020).

In addition, PGPR can promote plant growth by producing pathogen-antagonistic substances and/or by inducing systematic resistance in plants to pathogens (Glick, 1995). Recent research has also found that volatile organic compounds (VOCs) produced by PGPR play a significant role in promoting plant growth and provoking induced systemic resistance (ISR; Kanchiswamy et al., 2015; Raza et al., 2016). ISR against pathogens – enhanced plant defense ability, provoked throughout the entire plant – is a mechanism used by PGPR to enhance the pathogen resistance of their host plant (Beneduzi et al., 2012). Development of systemic resistance is indirect but a key strategy to prevent biotic losses in crops; beneficial PGPR can trigger ISR in plants (van Loon et al., 1998; Beneduzi et al., 2012) upon colonization of plant roots, as can mycorrhizal fungi (van Loon et al., 1998; Pozo and Azcón-Aguilar, 2007; Pérez-de-Luque et al., 2017). ISR establishes a primed state by inducing more rapid defense-responses upon pathogen attack (Mauch-Mani et al., 2017). ISR has been identified and illustrated in many plant species and has been found effective against a broad spectrum of plant pathogens, including bacteria, fungi, viruses, and even against herbivorous insects (van Loon et al., 1998; Bhattacharyya and Jha, 2012). Traditionally, ISR was thought to be very distinct from systemic acquired resistance (SAR), which is induced by local infection of pathogens and involves salicylic acid (SA) signaling and expression of pathogenesis-related (PR) protein genes (Pieterse et al., 2009; Gao et al., 2015). Unlike SAR, ISR results in the expression of defense-related genes that are JA- and ET-responsive (Mathys et al., 2012), and do not necessarily involve accumulation of PR proteins. However, increasing evidence suggests that the signaling pathways differ depending on the type of PGPR, pathogen, and host plant. ISR-inducing PGPR that use the SA-pathway, and not the JA/ET (ISR) pathway, were reported in past studies (Maurhofer et al., 1994, 1998; De Meyer and Höfte, 1997; Audenaert et al., 2002; Barriuso et al., 2008; van de Mortel et al., 2012; Takishita, 2018). As other plant hormones, such as gibberellins (Navarro et al., 2008), auxins (Kazan and Manners, 2009), cytokinins (Giron et al., 2013), and brassinosteroids (Nakashita et al., 2003) have also been demonstrated to function as modulators of the plant immune signaling network, hormone crosstalk is believed to exist, providing plants with capacity to finely tune immune responses for their growth and protection (Pieterse et al., 2014). Elucidating the detailed molecular mechanisms underlying the PGPR-induced ISR is an important challenge for future research (Bulgarelli et al., 2013; Turner et al., 2013; Zhang et al., 2013; Müller et al., 2016; Zgadzaj et al., 2016; Kruitwagen et al., 2018; Lommen et al., 2019).

PGPR are involved in diverse mechanisms to enhance plant growth and/or act as BCAs. To ensure the sustainability and cost-effectiveness of agricultural systems, crop production promotion and pathogen control could be considered together. It would be very efficacious to identify and develop novel, effective PGPR strains providing several beneficial activities, such as improving nutrient uptake, improving stress tolerance, enhancing plant growth, and combating fungal or bacterial pathogens. In particular, it seems possible to identify and develop PGPR that both suppress plant disease and more directly stimulate plant growth, bring dual benefit. It seems likely that phytomicrobiome members that have coevolved with plants for long periods of time and perform one activity beneficial to plant growth very well, will perform other plant-beneficial activities. This is in the best interests of the microbe.

## Challenges in Using PGPR As Biocontrol Agent

The goal of PGPR-based biocontrol is to provide alternative and sustainable approaches for disease management. The United States and Europe have become the largest potential markets for biocontrol products, followed by South America (Barratt et al., 2018). A large set of PGPR have been studied at the laboratory scale and some of which have been commercialized (Glick, 2012; O’Brien, 2017; Rosier et al., 2018). In the last decade, there has been continued growth in the commercialization of BCAs (Fravel, 2005; Bashan et al., 2014; Mishra et al., 2015; Begum et al., 2017; O’Brien, 2017). A number of research projects are now focused on developing novel biocontrol products for Europe^1^ and the United States.^2^

While the BCA market is expanding, it is still far from being broadly applied, and the use of chemical pesticides is still dominant in crop production (Mishra et al., 2015). There are important challenges that need to be overcome before biocontrol practices can be widely accepted and optimally utilized (Bashan et al., 2014).

Multifaceted research is required to improve our understanding of the efficacy of BCA in control of specific diseases. Ideally, a BCA should include as many beneficial features as possible, for example, growing rapidly *in vitro* for the purpose of commercial production, possessing high rhizosphere competence, enhancing plant growth capabilities, having a broad spectrum of bioactive metabolites, being environmentally safe, being aggressively compatible with other rhizobacteria, and being tolerant to abiotic stress (Nakkeeran et al., 2005; Egamberdieva and Lugtenberg, 2014; Ilangumaran and Smith, 2017; Lyu et al., 2020). Effective root colonization is an important feature of good biocontrol PGPR as only when effective strains colonize the rhizosphere and/or root tissues, can they perform their action against pathogens. Inconsistent performance of the inoculated PGPR due to poor survival in soil, compatibility with the crop, interaction with indigenous microbial organisms and other environmental factors (Martínez-Viveros et al., 2010; Vejan et al., 2016) is a major barrier that prevents the wide application of BCA. For researchers, survival and colonization should be considered as essential characteristics during the identification of useful isolates.

The effect of bacterial isolates against targeted pathogens is normally determined by *in vitro* antagonism tests, as a first step, followed by greenhouse and field research trials (Bashan et al., 2014). The consistency of performance is generally evaluated at various geographical locations, under a range of climatic conditions. Furthermore, because rhizobacteria have strong relationships with host plants, crop species are also considered (Choudhary et al., 2011). Monitoring of BCA development in soil also helps in understanding their subsequent endurance and interactions with host plants. Development of BCA under greenhouse conditions is easy to monitor, due to relatively stable environmental conditions. Abiotic stress could also be considered in this stage, investigating performance under various climate change scenarios. Experimental testing in the greenhouse is valuable not only for providing practical guidance regarding application of BCAs for controlled-environment crop production, but also for providing theoretical support for field application.

Stability of PGPR products is influenced by the production procedure, formulation, transportation, and storage conditions. Thus, following the identification of potentially deployable microbial strains, there is a need to achieve subsequent high levels of survival of biocontrol products (McIntyre and Press, 1991), improve formulation of the novel technology (Lobo et al., 2019), and achieve desirable attributes such as long-term shelf life (Carrasco-Espinosa et al., 2015). During the production procedure, diverse culture media and growth conditions have been studied and optimized for production of targeted microbial types (Pastor-Bueis et al., 2017; Khanghahi et al., 2018; Zhang et al., 2019). Achieving low-cost production at large scale is a difficult task, but reachable with careful research effort (Trujillo-Roldán et al., 2013; Carrasco-Espinosa et al., 2015; Kang et al., 2017). Liquid formulations are the most popular forms (Lee et al., 2016); many researchers tried to increase the shelf life of PGPR through lowering storage temperature and/or modifying the combinations of additives (Arriel-Elias et al., 2018; Berger et al., 2018).

Apart from the problems mentioned above, comprehensive analyses of risks and benefits of application of BCAs are also required since agricultural decisions for choosing a disease control strategy are made on the basis of this balance. As the modes of action of PGPR are diverse, the identification, performance assessment, and registration of potential strains take time and require academic and industrial sector support.

On the other hand, the use of natural sources for combating pathogens also poses legal and ethical challenges of its own, due to the fact that exotic BCAs can threaten local biodiversity (De Clercq et al., 2011; Simberloff, 2012; Hajek et al., 2016), leading to restrictions on the importation of new species/populations into some jurisdictions. In this regard, it is easier to utilize PGPR in the protective conditions of a commercial greenhouse which provide a relatively isolated and controlled environment, as compared to field production, while potentially having less negative effects of the ecosystem.

Regulatory issues are another barrier to global application of PGPR-based biocontrol. At the moment, each individual country has its own regulatory system and the nature of these varies considerably among countries (Bashan et al., 2014). For instance, it was indicated that barriers to growth of the BCA industry in Australia included high costs of developing new commercial BCAs (Begum et al., 2017). One of the leading factors is the high regulatory costs in terms of importation of new BCAs into Australia (Begum et al., 2017). Registration of BCAs requires close cooperation between governmental agencies and academic plus industrial sectors, to support the much needed evaluation and marketing of these new products. An obvious problem is the lack of programs for demonstrating the financial and environmental benefits of their utilization (Heimpel et al., 2013). Commercialization should follow international legislation for international markets and local practical uses. An International Organization for Biological Control (IOBC)^3^ has gathered together practitioners and researchers from widely diverse fields to promote the identification of any limitations to broad-application of biocontrol products and to provide recommendations for mitigating these limitations (Barratt et al., 2018).

Furthermore, at the grower level, low adoption of commercial BCAs occurs, as crop producers who have not been engaged in biocontrol or lack of basic knowledge of the area may see only slow progress in general acceptance, or no initial impact on crop disease within their production systems (Begum et al., 2017). Thus, farmers may have little or no financial benefit compared with pesticides which tend to be more reliable and predictable. Associated programmed introduction and local workshops could help to promote distinct BCA applications in specific farming areas.

In summary, PGPR-based biocontrol shows great promise, reducing the reliance of agrochemical in crop production. Wider application of PGPR biocontrol products requires substantial evidence of efficacy and acceptability, evidence of which must be provided not only to the regulatory agencies, but also to crop producers, to gain their confidence in the capacity of the new product, in terms of disease control and yield improvement. High-valued crop production in the greenhouses could be an excellent place for utilizing PGPR as BCAs, and exploring the efficacy under a variety of abiotic stresses. Based on current successful examples in greenhouse trials, the advantage of controlled environment conditions would be and easier venue for initial development and implementation of BCA for disease management and attendant crop plant growth promotion.

## Author Contributions

XJ and YT gathered reading material and wrote the review paper. GZ provided perspective and editorial input. DS provided intellectual context, guidance in scientific knowledge, and editorial input. All authors contributed to the article and approved the submitted version.

### Conflict of Interest

The authors declare that the research was conducted in the absence of any commercial or financial relationships that could be construed as a potential conflict of interest.

## Footnotes

^1^https://cordis.europa.eu/project/id/612713/reporting

^2^https://www.aphis.usda.gov/aphis/ourfocus/planthealth/plant-pest-and-disease-programs/biological-control-program

^3^http://www.iobc-global.org/index.html
